# Conceptualising metabolic disorder in Southern Africa: Biology, history and global health

**DOI:** 10.1057/s41292-018-0122-3

**Published:** 2018-06-20

**Authors:** Megan Vaughan

**Affiliations:** 1 Institute of Advanced Studies, University College London, Gower St, London WC1E 6BT, UK

**Keywords:** Metabolic disorder, Non-communicable disease, Africa, Malawi, Epidemiologic transition

## Abstract

This paper traces the history of the concept of metabolic disorder in global health and its application to the collection of health metrics relating to the ‘epidemic’ of non-communicable diseases in Southern Africa, with a focus on Malawi. Although the contemporary science of metabolism points to complexity and contingency, the application of a simplified version of ‘metabolic disorder’ or ‘metabolic syndrome’ as the supposed central driver of non-communicable disease in low- and middle-income countries runs the risk of obscuring the ways in which local circumstances and histories interact with global forces to produce epidemiological change. The paper discusses health data collection and its interpretation in Malawi to demonstrate how the use of this concept has led to a narrowing of the category of non-communicable disease and a neglect of the role of infectious disease in producing chronic conditions. Finally, the paper points to alternative approaches which might yield a better understanding of pressing health problems.

All over the world, and particularly where laboratory conditions are in short supply, the measurement of the waist circumference has come to be used as an indicator of susceptibility to ‘metabolic syndrome’ or ‘metabolic disorder’: a set of conditions including obesity, raised blood pressure, raised triglycerides, reduced HDL cholesterol and raised plasma glucose levels. These conditions in turn indicate susceptibility to heart disease, diabetes mellitus, stroke and other serious health consequences. Although officially the definition of metabolic syndrome requires that an individual display central obesity *and* at least two of these other indicators, in some circumstances the waist measurement comes to stand in for all of them. It only requires a tape measure, and anyone can do it.

The tape measures are out in parts of Africa where expanding female waistlines are causing alarm (Okafor 2012; Tuei et al. 2010). Consider this scenario. A woman in her 30s in a rural area of Malawi is having her waistline measured by a nurse in a local clinic. She is told that her waist measurement is too large and that this indicates that she may be vulnerable to a range of conditions from heart disease to Type 2 diabetes. The country is experiencing what some clinicians regard as a new ‘epidemic’ of non-communicable diseases including a range of conditions glossed as ‘metabolic syndrome’ or ‘metabolic disorder’. The marked rise of adult female obesity seems to be both symptom and cause of this new set of health concerns in a country more accustomed to dealing with the effects of under-nutrition, malaria, HIV/AIDS and other communicable diseases. The Malawian public has long been educated on the problems caused by under-nutrition, but overweight and obesity are apparently new phenomena. The nurse must educate people on these matters. She informs the woman in front of her that though her mother may have told her that being a bit fat is good and healthy and a kind of insurance again dearth and disease, this is a mistaken idea. But 30 years earlier this same individual, in the same clinic, was a small baby having her upper arm circumference measured by another nurse. The upper arm measurement like the waist measurement was a proxy for something—in this case severe infant under-nutrition. The woman’s mother was told that the baby was too small, and was vulnerable to all sorts of diseases and might never grow up properly, so it was important that she be fed with additional supplementary foods until she had ‘caught up’.

What join these two stories are not only the tape measure, a ‘syndrome’ and the international creation of norms, but an actual life history and perhaps an earlier life history, and a longer-term economic, political and environmental history. This paper is a preliminary exploration of how the concept of metabolic syndrome is being mobilised in parts of southern Africa in the context of rising rates of non-communicable disease understood as part of an ‘epidemiological transition’. Waistlines, and the female waistline in particular, have become an object of measurement and a prognostic sign of the rise of ‘diseases of lifestyle’ which pose a significant challenge to the region’s fragile health systems. In this paper, I first outline the evolution of this biomedical concept, its relationship to the science of metabolism and to ideas about temporality and change, in particular the theory of epidemiologic transition. Next I turn to look at how this biomedical concept has recently informed the collection of health statistics in one country in southern Africa, Malawi, and is determining understandings of the rising incidence of non-communicable disease in this context. I argue that though the evolving science of metabolism points to complexity in the relationship between biology, environment and history, ‘metabolic syndrome’, or ‘metabolic disorder’ by contrast, is employed in a reductive fashion that reduces complex epidemiological change to a set of ‘global risk factors’ that focus on individual behaviour and lifestyle. This approach not only obscures the important relationship between infectious and non-communicable disease in Malawi, but also obscures the historical processes at work in that context. Finally, I briefly refer to alternative frameworks for understanding the rise in non-communicable and chronic conditions in Malawi.

## Metabolic Knowledge

The idea of the ‘metabolic syndrome’, as employed in the context of the ‘global north’ speaks to a sense in which the contemporary body is exhibiting a disorderliness that threatens self-destruction. Science studies scholar Hannah Landecker has dubbed this “post-industrial metabolism” (Landecker 2013a). In a series of illuminating papers, Landecker traces the history, and philosophy of the science of metabolism since the late eighteenth century, including the implications of recent work in genetics and epigenetics for our understanding of the ways in which metabolism in conceived (Landecker 2011, 2013a, b). She has shown how productive the science of metabolism has been in the last 200 years, and how it has both reflected and contributed to our understanding of the relationship between the body, environment and political economy. Although the concept of ‘metabolic syndrome’ as it evolved in the twentieth century clearly derives from changing understandings of metabolism itself, it is also closely related to the development of population level studies and new statistical techniques, which are now being applied to the collection of global health data. In the context of low and middle-income countries now experiencing an increase in non-communicable disease, ‘metabolic syndrome’ provides a universalising framing device for the collection of data on these conditions, which allows global comparison, and an implicit (sometimes explicit) diagnosis of their causation as one of ‘lifestyle’ change. In the institutions of global health and their data collection arms, ‘metabolic syndrome’ appears to describe a disorderly and speeded-up process of ‘epidemiologic transition’.

The concept of metabolism, from its inception, has spoken to large economic, environmental and social processes. The modern biology of metabolism is usually traced to the work of mid-nineteenth century French physiologist, Claude Bernard, who famously observed that a ‘dead’ animal liver could manufacture sugar.^1^ As Landecker and others have demonstrated, in industrialising nineteenth century Europe the body was generally understood in terms similar to that of the newly invented combustion engine. The environment provided fuel in the form of food to the animal organism which it broke down, allowing energy to be expended. The leftover waste was expelled. But Bernard’s experiments led him to elaborate the idea of a nutritive reserve—animals did not simply ingest and break down food in a linear way, they also engaged in a translation process which converted alien substances into new matter. Just in order to exist, all organisms had to maintain themselves and build up nutritive reserves, producing a stable internal environment in the face of changing external conditions. It is of course not difficult to see connections between Bernard’s biological theories and the contemporaneous concerns of the industrialising European state. The concept of metabolism both fed on and produced meaning around the relationship between the body, the individual and society (Nally 2011). Landecker argues that the idea of a nutritive reserve enabled the individual body to be figured as autonomous, able to withstand fluctuations in its surrounding environment (Landecker 2013b).

Although ripe with metaphoric possibilities to describe relations between body, individual, society, political economy and nature, metabolism in the late nineteenth century was not ‘merely’ a metaphor; rather, it was a concept that described the actual energetic and material exchanges between societies and their natural environments. Amongst other thinkers, Karl Marx worked with metabolism as a physiological concept, mediating between man and nature (Arendt 1958). Twentieth century advances in biochemistry moved metabolism further inwards to the exchanges and conversion work of cells and tissues which enabled the stable state of the body to be achieved. But, as Landecker has shown, if the concept (and the body it described) had apparently reached a stable state in the twentieth century, it was set into motion and flux again in the twenty-first, both by advances in genetics and epigenetics which link ingestion with heredity and reproduction, and by a global epidemic of ‘metabolic disorders’ speaking to an apparent inability of our individual bodies to maintain a steady internal state. In a situation in which ‘chemical landscapes’ can now be seen to drive microbial evolution and in which food as ‘exposure’ can have epigenetic consequences, the new concepts of metabolism are marked by radical porosity as well as overlapping temporalities (Landecker 2011; Landecker 2013a; Grote and Keuck 2015; Solomon 2016).^2^

Although the evolution of the biomedical concept of ‘metabolic syndrome’ is clearly dependent on the developing science of metabolism, it also has a sociopolitical life of its own. In his account of the racialisation of metabolic syndrome in the USA, Anthony Ryan Hatch traces the technical developments which contributed to the establishment of ‘metabolic syndrome’ in the twentieth century, including the elaboration of population level studies and the employment of the predictive power of statistical techniques (Hatch 2016, chapter 2). The French physician, Jean Vague, first identified a causal relationship between obesity, heart disease and diabetes in the 1940s and the specific term ‘metabolic syndrome’ first appeared in biomedical literature in the 1970s and has been widely used since. For the last 10 years or so, the concept of metabolic disease or syndrome has been increasingly used as a framing device in the literature on the growing prevalence of non-communicable diseases in low- and middle-income countries. Its employment has not been without controversy.

Despite the efforts of the World Health Organisation to standardise its definition, there continue to be struggles over its meaning and application, particularly as these effect societies of the ‘global south’. At the most reductive these struggles concern the fraught question of waist measurement, with which I began this paper (see World Health Organisation 2008a). By the 2000s, it was becoming clear that the variation in diagnostic criteria for metabolic syndrome being used by different organisations (notably the WHO, the American Heart Association and the International Diabetes Federation) was causing confusion and casting doubt on the validity of the concept itself. In 2009, a Joint Statement was issued that attempted to harmonise the criteria used globally to define the syndrome (Alberti et al. 2009). Although there was general agreement on most criteria, including raised blood pressure, glucose levels and dyslipidemia, and a single set of ‘cut-off’ points for these measures was agreed, the waistline measure proved a sticking point.^3^ Here international health diplomacy appears to have broken down, with the Europids (represented by the American Heart Association) holding out for a universally higher cut-off and the International Diabetes Federation arguing for ethnically specific waist circumferences. In the end it was agreed that more work was needed on the waistline measure and in the interim national and regional cut-off points were established.

Behind this discussion lies a longer and larger history. The waistline issue is just one instance of the tension that runs through much global health policy and literature between universalising narratives and definitions on the one hand and an insistent attention to (and sometimes reification and racialisation of) difference on the other hand. Behind these, rarely acknowledged, trail colonial and racial histories. Three recent social anthropological studies of metabolic syndrome and obesity illuminate the international, national and ethnic and gender politics of measurement. Harris Solomon describes the negotiation over international norms in India in relation to the Body Mass Index (BMI) and the experience of metabolic diseases, doubt having been cast on the usefulness of BMI measures with the ‘discovery’ that outwardly ‘thin’ Indians could be as metabolically unwell as outwardly ‘fat’ Europeans and North Americans (Solomon 2016). In rural Guatemala Emily Yates-Doerr similarly explores the meaning of BMI, body shape and nutritional advice in the context of a rapidly changing and industrialising food economy. Although rural Guatemalan women are well aware of the health dangers posed by new food regimes, they also value a degree of abdominal fat and point out that their inherited body morphology does not dispose them to narrow waistlines (Yates-Doerr 2015). Furthermore, even if convinced that their waistlines needed slimming, the food environment in which these women live is not one in which they are able to exercise ‘free choice’ in food consumption. Amy Moran-Thomas traces the effects of metabolic thinking through her nuanced study of the experience of chronic disease (particularly type 2 diabetes) in Belize (Moran-Thomas 2012).

Global health interventions are often criticised for imposing supposedly universal measures and norms that are, on closer inspection, derived from specific historical and cultural contexts. Not infrequently these are, in effect, European or North American norms, and explanations for divergence are often racialised. This is true in the field of metabolic disease where the causes of ‘black’ diabetes and heart disease or ‘Mexican’ diabetes have been sought in hypothesised genetic origins, often reinscribing race and diverting research away from more immediate factors such as economic and social inequality or discrimination (Hatch 2016; Pollock 2012; Montoya 2011; Whitmarsh 2008). There are, therefore, many good reasons to be suspicious of a concept such as metabolic syndrome as interpreted in the context of a ‘liberalised’ global health industry closely associated with genomic and pharmaceutical markets. Vague, descriptive, somewhat messy and yet armed with apparently scientifically validated measurements, it also falls squarely within the realm of an American (but increasingly global) model of medicine characterised as ‘treatment as prevention’, subject of incisive critique by Joseph Dumit, amongst others (Dumit 2012). This neoliberal model of health, which has thrived in the context of the growth of chronic conditions, not only ‘feeds’ the pharmaceutical industry but also places responsibility for outcomes on the patient who, armed with a blood pressure or sugar monitor (and increasingly with smartphones and apps) and medications, can both anticipate and manage their own life-long conditions (Whitmarsh 2013; Glasgow and Schrecker 2015). This is a deeply problematic model in and of itself, but all the more so when applied in a context of failing or unaffordable professional medical provision, as is often the case in low- and middle-income countries. In the case of southern Africa, many populations are already ‘primed’ for this kind of intervention by their experience of the management of HIV/AIDS and the development of what has been termed “therapeutic citizenship” (Nguyen 2010). Pharmaceutical companies are not simply waiting in the wings of the emerging ‘epidemic’ of metabolic syndrome in the global south, they are arguably, helping to create it. In circumstances (such as much of the African continent) in which reliable longitudinal data are absent, it is possible to create knowledge where none existed before.^4^

The globalisation of the metabolic syndrome as framing device and prognostic diagnosis has emerged in the wider context of the increasing attention paid by global health actors to the rise in prevalence of non-communicable diseases in the global South (Reubi, Herrick and Brown 2016; Glasgow and Schrecker 2015). Beginning in the 1980s, but gathering speed in the 2000s, international organisations, including the WHO, began to express anxiety over the apparent threat posed by increasing rates of ‘NCDs’ (particularly cardiovascular disease, cancer, diabetes and chronic respiratory disorders) in low- and middle-income countries.^5^ By 2010, an increasing volume of epidemiological surveillance data was revealing that of the 60% of global mortality caused by these conditions, 80% was taking place in low- and middle-income countries. In many of these countries in Asia and South America, NCDs were becoming the leading cause of death. Only the African continent, with its persistent infectious disease problems lagged behind, but Africa was also fast catching up (Okafor 2012). Although to some extent the very rise in incidence of NCDs in the global south must be seen as a result of the successes of ‘development’ (rising living standards, reduced deaths from infectious diseases and ageing populations), nevertheless, as David Reubi and others have argued, it has tended to be viewed with alarm by policy makers (Reubi et al. 2016). Often this alarm is expressed in explicitly capitalistic and global security terms: the economic burden of long-term conditions and rising rates of disability associated with them threaten the future ‘development’ of low- and middle-income countries on which the global economy depends (Daniels et al. 2014). But there are other reasons for concern, including the knowledge that African health systems are for the most part very poorly equipped to deal with long-term conditions. The ‘cost’ in terms of human suffering for individuals and their carers of a late diagnosis of cancer, or the complication of diabetes and stroke is not easily described by the usual health metrics (Livingston 2012; De-Graft Aikins et al. 2012; Tagoe 2012; Whyte 2015).

The available evidence shows that in sub-Saharan Africa as a whole “the leading cause of total premature mortality and disability is not cardiovascular disease, diabetes, or other non-communicable disease but rather communicable, maternal, neonatal an nutritional causes and their related risk factors” (Mensah 2013, p. 246). However, variation across the continent is marked, and southern Africa appears to be leading the way in what is sometimes described as a coming ‘tsunami’ of non-communicable disease. Given that, for many regions, the ‘tsunami’ has not yet arrived, policy makers are understandably interested, not only in current rates of NCDs, but also in the ‘risk factors’ which have been shown to predict future increases in NCD incidence elsewhere. These ‘traditional’ risk factors include a rise in BMI and fasting plasma glucose as predictive of future diabetes and cardiovascular disease; rising systolic blood pressure as predictive of stroke and other cardiovascular disease and a rising number of persons with at least one of the combined risk factors of tobacco smoking, poor vegetable and fruit consumption, low levels of physical activity and excessive alcohol consumption. ‘Metabolic syndrome’ comes in here as shorthand and as a tool of prognosis. These risk factors may not appear to explain current epidemiology but, it is assumed, they are predictive of future trends.^6^ For example, the profile of cardiovascular disease in sub-Saharan Africa is ‘unique’ amongst regions of the world with around half of these cases due to causes other than atherosclerosis, and the age of death is younger. Stroke was the leading cardiovascular cause of death and disability in sub-Saharan Africa in 2010, and the role of infection and inflammation as a contributory factor in CVD epidemiology is also recognised (Moran et al. 2013). But the comparative and historical analysis of other regions of the world leads to the conclusion that CVD patterns will increasingly come to resemble those of more ‘developed’ regions. This is where ‘transition theory’ comes in.

## Historical Frameworks and Temporalities: Transition

If metabolic syndrome is related to the category of ‘NCDs’ as a framing device through which changes in global epidemiology are now viewed, the more general model from which both derive is that of the ‘epidemiologic transition’, first put forward by the Egyptian demographer, Omran, in the early 1970s as an adjunct to demographic transition theory (Omran 1971). This influential account of the epidemiological consequences of modernisation proposed that all parts of the world would eventually move through the ‘transition’ experienced by industrialised ‘developed’ countries. The latter, having ‘conquered’ the majority of infectious diseases, were characterised by high life expectancy, low fertility rates and ageing populations, with a consequent disease profile that Omran characterised as the “Age of Degenerative and Man-Made Diseases”. “Underdeveloped” countries, Omran argued in 1971, still laboured in the “Age of Pestilence and Famine”, characterised by high fertility and mortality rates, young populations and agrarian economies. The theory has been subject to many critiques, revisions and qualifications over the intervening decades. Indeed, in recent years some historians of Britain and other parts of Europe have cast doubt on its usefulness for understanding epidemiological change in the historic ‘heartlands’ of industrialisation which provided the model for global change (Mercer 2014). Nevertheless, the idea of an epidemiological transition remains remarkably resilient, and continues to appear in policy literature.^7^

In the case of the African continent, the unevenness of demographic change and the persistence of communicable disease (despite major health improvements) means that the ‘transition’ to an epidemiological regime characterised by the dominance of non-communicable disease is highly uneven, both spatially and temporally. In southern Africa, the impact of the HIV/AIDS epidemic since the mid-1980s has been profound. To regard this as a mere ‘bump’ in the ‘transition’ would appear to stretch the model beyond plausibility. In recognition of this, some experts refer to the sub-Saharan African region as experiencing a ‘double burden’ of disease composed of communicable diseases *and* an increasing incidence of non-communicable disease (Whyte 2012; Marshall 2004). This ‘double burden’ has multiple dimensions. As treatments for HIV/AIDS have become more effective and more widely available, so HIV infection is now often viewed as a chronic long-term condition, producing a range of inflammation associated or immunodeficiency related illnesses including cardiovascular disease and certain cancers (Deeks et al. 2013). Anti-retroviral therapies themselves may be contributing to this picture. Co-infection between HIV and TB has for some time been recognised as a significant dimension of the AIDS epidemic in southern Africa but increasingly patient experience is one of complex multi-morbidities: one body experiencing simultaneously a set of conditions which have traditionally been viewed as belonging to different ‘stages’ in epidemiological histories. For example, infection with tuberculosis appears to predispose patients to diabetes, and the converse has also been noted (Jeon and Murray 2008; Nyirenda 2016). What this complex picture of co- and multi-morbidities means for clinical practice and patient experience has been vividly described by Julie Livingston (Livingston 2012) and in an emerging literature on southern Africa informed by the concept of ‘syndemics’ (Mendenhall et al. 2015; Mendenhall and Norris 2015).

If metabolic syndrome as an object of analysis in southern Africa came into being within a more general concern with the increased incidence of non-communicable diseases, this was also a context marked by an epidemic of a communicable chronic disease in the shape of HIV/AIDS. Despite this specific context, the idea of ‘transition’ from an infectious disease regime to one dominated by non-communicable chronic conditions has remained highly influential in accounts of the changing epidemiology of southern Africa. Although transition theory is a universalising account, apparently paying little attention to difference, it is also an evolutionist account of modernisation and in this sense an inheritor of a history of racialised thinking on disease distribution that dominated southern Africa in the twentieth century. The processes of urbanisation and ‘modernisation’ have long been regarded as pathological in this region, and the history of epidemiological thinking here is inseparable from a labour migrant economy which figured African workers as having rural, tribal bodies and minds easily unsettled by exposure to ‘civilisation’ (Vaughan 1991). Harris Solomon has noted a similar colonial residue in the epidemiological imagination in India where diseases of ‘modernity’ (and their sufferers) are regarded as ‘poor copies’ of their western counterparts (Solomon 2016). Of course, not all epidemiological analyses of the consequences of the rapid economic and social changes experienced in the global south are so evidently neo-colonial. In South Africa a powerful tradition of social medicine has existed since the 1930s and has contributed to a more radical version of the ‘transition’ story, one that stresses the health consequences of stark inequalities, rapid urbanisation and globalisation. In the study of the rising incidence of non-communicable diseases, this has resulted in important research on, for example, the consequences of the globalisation of the food and agriculture industries as they contribute to the problems of obesity, diabetes and the metabolic syndrome.^8^ Incisively critical as this work is, much of it still relies on a historical framework of ‘transition’ and an assumption that the consequences of globalisation and late capitalism for the bodies of those in the ‘developing world’ can be modelled on the basis of the European and North American experience of industrialisation. Although there are good reasons for assuming some common consequences of, for example, globalised urban diets, there are equally important indicators that context and history (beyond ‘transition’ or ‘race’) matter (Solomon 2016, p. 228; Hatch 2016; Pollock 2012; Lock 1993; Lock and Nguyen 2010). The need to understand past histories and particularities is now also being recognised by medical researchers in the southern African region (see, for example, Nyirenda 2016). The countries of the ‘global south’ are both at the receiving end of metabolic science and are producing the knowledge which informs its transformation.

In the next section of this paper, I turn to Malawi to explore the ways in which new metabolic knowledge has been created in the past 15 years or so. I examine how a narrow range of global ‘risk factors’ for metabolic disorders has come to stand in for a more complex set of epidemiological and demographic changes and processes.

## Creating Metabolic Knowledge in Malawi

In June 2016, the Al Jazeera news network broadcast a feature on food shortage in Malawi. The seasonal rains having failed in some parts of the country (attributed by many to the El Nino effect), health facilities in the southernmost part of the country were already experiencing a marked increase in infant and child malnutrition. The programme showed women waiting in a mother and child clinic in the Lower Shire Valley, many of them holding babies limp in their arms and visibly severely malnourished or wasted. As they waited their turn for medical attention, a government nutritionist was using the opportunity to give the women dietary advice. Diversity of diet, he was saying, is very important. The standard Malawian main meal consists of a maize porridge (*nsima*) and an accompanying relish (*ndiwo*) that for the majority of rural people comprises vegetables and legumes and less regularly, fish and meat. The dietician was stressing the importance of choosing the relish carefully to maximise dietary diversity—you have your *nsima*, he was saying, now, will you have meat or fish or beans to go with it? His talk was accompanied by a plate-based coloured chart showing the major food groups. The women appeared to be listening attentively, but when the dietician elaborated on the importance of the choice of relish, one woman began to laugh and the laughter spread through the whole waiting room. The dietician smiled and looked a little embarrassed. As he knew only too well from the evidence in front of him, these were women struggling to find any food at all with which to feed their families—dietary diversity was not currently at the top of their agenda.

Misdirected as the dietician’s advice may have been in the clinic, dietary diversity (or the lack of it) is a real issue in Malawi as, it appears, is obesity. In the same week in June 2016, the director of one of Malawi’s major hospitals was telling me about the growing problem of type 2 diabetes and obesity. Recently, he said, he had had a visit from officials of the government’s Home Affairs department who had asked him what could be done about the fact that many policemen were now so overweight that they were unable to perform their job effectively. In fact I already knew about this as the Malawian press had picked it up as a story with the headline “Policemen too fat to chase thieves!”. Hardly a day goes by in Malawi without the media reporting some aspect of the country’s newly discovered ‘epidemic’ of type 2 diabetes and its relationship to changing diets and ‘lifestyles’. As in other parts of the continent (Whyte 2015), talk of “sugar” and “pressure” has become common as the “hidden” conditions of diabetes and hypertension are made visible through the metrics of glucose tests and blood pressure monitors. This is despite the fact that Malawi is a poor country with lower rates of urbanisation than others in the region, and one characterised by periodic serious food crises and an improving but still high level of chronic under-nutrition, particularly in infancy. The situation in Malawi in 2016 seems to be characterised by the co-existence of under-nutrition and obesity, a phenomenon noted for other ‘low and middle-income countries’.

The fact that “sugar” and “pressure” have been added to a local medical lexicon in Malawi is in no considerable part due to the increasing attention paid to non-communicable disease and diseases of metabolism by global health institutions, and particularly by their data collecting agents. This does not mean that people in Malawi were not *experiencing* the consequences of these conditions before they were being measured and monitored, but their new meanings are now firmly located within a biomedical account of the rise of non-communicable disease incidence and are mediated by a set of biotechnologies. As in Uganda (Whyte 2015), the local discourse on type 2 diabetes (and other conditions) appears to be centred on an idea of mal-adaptation of local bodies to new lifestyles, resonating with evolutionary perspectives on contemporary disease patterns and with colonial thinking. There is a certain irony in the fact that while critical social and historical analyses of contemporary Africa generally avoid the tropes of ‘modernisation’, public perceptions are often focused on this very concept.

Epidemiological survey data have been central to the creation of this understanding of an apparently ‘new’ set of health concerns, coming on top of a still significant burden of infectious disease. The Malawian population is used to being surveyed, medically, socially and sexually (Biruk 2018). A small and accessible country with enduring problems of poverty, the country has come to exemplify many of the problems of ‘development’ in Africa and has been the site of a great many interventions, not all of which have succeeded in achieving their goals. In the global development and health community, this has led to an emphasis on ‘evidence-based’ policy and on the improvement in quality of that data.^9^

In Malawi, as elsewhere, the collection of health statistics informing the creation of biomedical and epidemiological knowledge has been heavily influenced by international organisations, particularly the World Health Organisation. In 2008 the WHO, reflecting the increasing visibility of non-communicable disease in low- and middle-income countries, published an Action Plan for the Global Strategy for Prevention of NCDs (World Health Organisation 2008b). As Sara Glasgow and Ted Schrecker have shown, this Action Plan, while explicitly acknowledging that NCDs are linked to trade, agricultural policy, taxation and heavily influenced by inequality, nevertheless ended by adopting an individualistic behavioural approach focused on the ‘global’ risk factors of tobacco use, unhealthy diet, physical inactivity and harmful use of alcohol (Glasgow and Schrecker 2015, p. 205). A recognition of the social determinants of health was sidelined by what critics have called “lifestyle drift” (Popay et al. 2010, p. 148). This “lifestyle drift”, coupled with a conception of “transition” reflecting Omran’s theory, is explicit the WHO’s 2010 Status Report which argues that “NCDs are caused, to a large extent, by four behavioural risk factors that are pervasive aspects of economic transition, rapid urbanization and 21st century lifestyles: tobacco use, unhealthy diet, insufficient physical activity and harmful use of alcohol” (World Health Organisation 2010, p. vii). This focus on supposedly modifiable risks factors came to frame the collection of evidence on NCDs in Malawi, redefining the very category of “non-communicable” disease in the process, and obscuring other questions which had been signalled in earlier (albeit inadequate) survey data and in some of the Global Burden of Disease estimates for the country.

The most immediate and direct outcome of the WHO approach to NCDS can be seen in the STEPS survey on “Chronic Non-Communicable Diseases and Risk Factors” which was carried out in Malawi in 2009 (Ministry of Health and WHO 2010). The STEP-wise approach to disease risk surveillance (STEPS) was devised by WHO as a standardised instrument for collecting and analysing data across member countries, thus enabling meaningful comparison and in-country monitoring. This approach has been employed to provide baseline data on the global status of non-communicable diseases and a number of surveys have been carried out in recent years in sub-Saharan African countries where such data are generally lacking.

There are three main components of a STEPS survey on non-communicable disease as outlined in the STEPS “instrument”. STEP 1 is a questionnaire designed to gather basic demographic and socio-economic data and to assess the prevalence of “risk factors”. STEP 1 includes “core” information which must be collected if the survey is to have validity, and “expanded” and “optional” items. Core items include the “behavioural” risk factors of tobacco use, alcohol use, fruit and vegetable consumption and physical activity, all widely recognised as “modifiable” risk factors for cardiovascular disease. STEP 2 of the survey consists of physical measurements, the “core” measures being weight and height, waist circumference and blood pressure and STEP 3 consists of biochemical measurements—the “core” ones being fasting blood glucose and total cholesterol.

The STEPs survey on “Chronic Non-Communicable Diseases and their Risk Factors” was carried out in Malawi in 2009 (Ministry of Health and WHO 2010). The executive summary of the report outlined the larger context of the report as relating to the increasing incidence, in low- and middle-income countries, of what used to be regarded “diseases of the West”, namely hypertension, heart diseases, stroke, cancer, diabetes, and chronic respiratory diseases. The authors pointed out that in Malawi data were scarce on the major risk factors, listed as tobacco smoking, overweight/obesity, excessive alcohol intake and physical inactivity.

The objective of the survey, therefore, was to measure these risk factors and also gather reliable biochemical data on the prevalence of hypertension, diabetes and raised cholesterol levels. A total of 5206 individuals aged 25–64 were surveyed, 67.5% of whom were women and local translations of the standardised STEPs questionnaire were administered, along with physical measurements and biochemical analysis. The results demonstrated that an astonishing 32.9% of the surveyed population had elevated blood pressure or were on medication for it—94.9% of who were unaware of their condition. The prevalence of diabetes mellitus was estimated at 5.6%. The raised cholesterol results were somewhat compromised by technical malfunctioning but it was concluded that 8.7% of the population had raised cholesterol levels, and that this was more common in women than in men. The gendered difference in many of the results was remarked upon by the authors of their summary, and was most notable in the results on “risk factors”. Smoking and potentially damaging alcohol consumption were overwhelmingly male, while overweight, obesity and physical inactivity were more common amongst women: 28.1% of women (vs. 16.1% of men) were judged overweight or obese and 12.6% of women (vs. 6.3% of men) were found to be insufficiently physically active. The dietary element of the risk factor analysis showed that though the surveyed population consumed sufficient vegetables, their fruit intake was inadequate. The authors noted that this latter result may have been affected by the fact that the survey was conducted in Malawi’s dry season when fruits are generally in low supply in rural areas. Although some risk factors (especially overweight and physical inactivity) were more common in urban areas, overall the authors concluded that the assumption that NCDs were an urban problem was a misconception: the Malawi survey indicated that the prevalence of raised blood pressure and diabetes was as high in rural areas as in urban ones (p. 28 of STEPS survey). Further detailed analysis of the results of the survey has been produced in a number of publications (Msyamboza et al. 2011, 2012a, b, 2013).

The 2009 STEPs survey would appear to have produced ‘hard’ data that can be used as a baseline for monitoring future change and for informing policy makers. Reliable data must be welcomed, and are surely better than the questionable estimations so commonly used in global health (Wendland 2016; Adams 2016). However, even the most comprehensive of these reports and surveys raise serious questions. In the case of the Malawi 2009 STEPs survey, technical issues, the low participation rates of some sections of the population and the sample size itself have all been pointed to as potential limitations (Crampin et al. 2016). Aside from these problems, there are more fundamental issues that arise out of the very process of standardisation and the assumptions that lie behind this. I focus here on two broad areas. The first is the way in which the underlying conceptualisation of NCDs as part of a ‘transition’ marked by the rise of ‘metabolic syndrome’ has decoupled them from the infectious disease context (Oni and Unwin 2015) and has also obscured the importance of other non-communicable and chronic conditions. The second is a similar separation from the continuing problem of under-nutrition and stunting in Malawi.

Analyses of existing (pre-STEPs) data on Malawi’s epidemiology show that, as with similar countries in the African region, in the early 2000s it was dominated by infectious disease and that young children still carried the largest disease burden and suffered the greatest mortality (Bowie 2006). Bowie’s (2006) account was based on WHO Global Burden of Disease estimates along with local data. The use of GBD estimates and calculations of Years Lost to Life (YLL), Years Lost to Disability (YLD) and Disability Adjusted Life Years (DALYs) have been widely questioned in the critical global health literature (Adams 2016). Nevertheless, Bowie concluded that the WHO estimates were reasonably in line with the data drawn from local survey sources such as the Demographic and Health Surveys (also standardised of course), censuses and HIV surveillance sites. The magnitude of the HIV/AIDS epidemic was graphically demonstrated, with the disease topping the lists as the major cause of mortality and of disability. Other major causes of mortality were lower respiratory tract infections, malaria and diarrhoeal disease (Bowie 2006). In a further set of estimates produced in 2013 by the Institute of Health Metrics and Evaluation, the leading causes of mortality were still HIV/AIDS, lower respiratory tract infection, diarrhoeal disease, protein-energy malnutrition and tuberculosis (The Commonwealth/IHME 2013).

The continued role of infectious diseases in the epidemiological profile of Malawi is important to note, not only because of the burden they continue to place on individuals, communities and health providers in their own right, but also because of the growing evidence for the relationship between infection and some chronic non-communicable conditions, as noted above (Nyirenda 2016). Although Malawi, along with many other countries in sub-Saharan Africa, has made huge advances in infectious disease prevention and treatment, not even the most optimistic of health experts would argue that the ‘era’ of infectious disease in Malawi has passed. HIV/AIDS remains an infectious disease, even though the wider use of anti-retrovirals means that it can also be accurately described as a chronic disease. Furthermore, the relationship between this infectious but chronic condition and non-communicable diseases such as Type 2 diabetes and cardiovascular disease is very likely to be a feature of Malawi’s changing epidemiological profile alongside urbanisation, ageing, changing diets and activity levels (Divala et al. 2016; Narayan et al. 2014; Levitt et al. 2011; Kumwenda et al. 2005; Bedell 2014). The role of infection is not confined to that played of HIV/AIDS. Tuberculosis (often associated with HIV/AIDS), diarrhoeal disease, lower respiratory tract infections and malaria all continue to feature prominently in Malawi’s disease profile. Tuberculosis has been associated with Type 2 diabetes, and malaria in early life may contribute to the development of cardiovascular disease (Dooley and Chaisson 2009; Woleneh et al. 2017; Moxon et al. 2014). The incidence of a range of cancers appears to be increasing in Malawi, as it is elsewhere in the region, but the leading cancers are those associated with infection: cervical cancer and Karposi’s sarcoma. Indeed, Malawi’s rates of cervical cancer are some of the highest in the world (Maseko et al. 2015; Rudd et al. 2017; Moses et al. 2017; Msyamboza et al. 2012a, b). The STEPs approach to non-communicable disease, with its focus on the risk factors associated with metabolic syndrome and its abstraction from the infectious disease environment underestimates the importance in the region of cancers associated with infection, and more generally obscures the role of infection in the aetiology of non-communicable disease.

The narrowing of the conceptualisation of non-communicable disease consequent on the focus on metabolic syndrome also erases a number of other conditions reported in earlier surveys. These include conditions that may have environmental causes not captured adequately by the concept of modifiable ‘lifestyle’ risk factors. For example, lower respiratory tract infections feature prominently in every survey of health conditions in Malawi and are a major cause of infant death. Indoor air pollution appears to lower resistance to respiratory infection and also contributes to the development of cardio and circulatory disease later in life (Fullerton et al. 2010). So significant is this factor that “household air pollution” was ranked as the leading risk factor for burden of disease in the 2010 Global Burden of Disease metrics (WHO/IHME 2010). Surveys from Malawi on self-reported and observed conditions revealed a range of painful and disabling conditions affecting mobility, and also what Bowie described in his 2006 review as “a huge burden attributable to psychological disease, although there is no local mental health survey to confirm this” (Bowie 2006, p. 110). This is a reminder of the more general neglect of the burden of psychological illness, both in its own right and as a co-morbidity of other conditions.

In principle there is no reason why the concept of metabolic syndrome should not include a consideration of under-nutrition, which remains a major problem in Malawi. The ‘double burden’ of malnutrition (including both under-nutrition and obesity) is well recognised in the global health literature, but the focus on overweight and obesity as a risk factor for NCDs and the divorce of infant and child health from adult health tend to direct attention away from the well-established theory of the developmental origins of disease (DOHAD) first put forward by Barker (Barker 2007; Wadhwa et al. 2009; Wells 2016). Over 25 years of research in this field has shown that foetal and early life insults (including malnutrition) might contribute to the development of a range of conditions in adulthood, including those commonly associated with metabolic syndrome, such as type 2 diabetes (Nyirenda 2016). This approach may be particularly relevant to Malawi. Despite considerable progress in infant survival and nutrition, rates of child stunting (widely thought to be indicative of future adult health) still hover around 40% (IFPRI 2014), and the country frequently experiences food shortages. Early under-nutrition, followed by rapid ‘catch-up’ growth (some of it induced by well-intentioned infant feeding programmes) may be one route, combined with environmental and dietary changes, to later susceptibilities (Wells 2016; Lelijveld et al. 2016). Furthermore, recent research in epigenetics points to the possibility that these effects may cross generations (Wells 2016; Wadhwa et al. 2009). As Landecker has shown, epigenetic research now positions food as “exposure”, shaping our physiologies and the ways in which our genes are expressed in our lifetimes and in those of our children and grandchildren (Landecker 2011). Although certainly not unproblematic in its application (see Pentecost and Ross 2018; Pentecost forthcoming; McNaughton 2011), the DOHAD approach and its epigenetic versions do at least have the merit of directing attention to the historical dimensions of epidemiological change and remind us that there is more to disease causation than individual lifestyle choice. It also reminds us that ‘lifestyle’ diseases are associated not so much with wealth (as the old label of “diseases of civilisation” implied) but with poverty and its reproduction within communities of the disadvantaged (Wells 2016).

## Conclusion

Having never ‘transitioned’ in the ways envisioned for them, there are many parts of the world whose metabolic complexity speaks to the highly uneven effects of modern capitalism, to colonial legacies and inequalities and to some of the unanticipated effects of well-intentioned global health interventions.

In her groundbreaking and heartbreaking account of the diabetes ‘epidemic’ in Belize, Amy Moran-Thomas has proposed that we reevaluate the conventional distinctions made between infectious and “noncommunicable” conditions and think instead in terms of “para-communicability” (Moran-Thomas 2012; see also Solomon 2016) and a different set of overlapping temporalities from that represented by ‘transition’. The staggeringly high rates of diabetes in Belize are not the result of ‘transition’ but of a sedimentation of disadvantage going back to the period of the slave trade, with ‘sugar’ as a constant theme. Few public health experts would go as far as to dissolve the category of ‘NCDs’, but the establishment, in 2015, of a *Lancet* Commission titled “Reframing NCDs and injuries for the poorest billion” is recognition that the current framing of these conditions is less than illuminating of their causes (and possible remedies) for the world’s poorest communities (Burkham et al. 2015; www.ncdipoverty.org). In many parts of the world, including Malawi, a considerable greater part of the NCD burden falls on the young and the poor and rural dwellers, and cannot be straightforwardly attributed to the ‘big four’ conditions and their ‘big four’ risk factors. Malawi is one of the countries taking part in the work of the Lancet Commission and in November 2016 the Malawi National NCDI Poverty Commission was launched to investigate the underlying patterns and causes of NCDs in the country (Cundale et al. 2017).

Already in Malawi new studies are emerging that should give us a better picture (though by no means a comprehensive one) of the actual incidence of non-communicable disease, beyond but including those framed within the terms of ‘metabolic disorders’ (Price et al. in preparation). These studies will also be able to link non-communicable disease to infectious disease, particularly HIV/AIDS and its chemotherapy, as well as to economic status, education and levels of urbanisation, a range of environmental effects and ageing (for example, Allain et al. 2017; Moses et al. 2017). Moving away from ‘transition’ to a more complex conceptualisation of the temporalities of epidemiological change also has the potential to give us a somewhat different understanding of what may indeed a creeping ‘epidemic’ of type 2 diabetes and other conditions.

There may be multiple routes to metabolic syndrome. The overweight urban police officer, leading a relatively sedentary life and eating a diet increasingly determined by a powerful global food industry, appears to be on one route. The young slim man in a rural district receiving treatment for HIV/AIDS may be on another. The young woman with whose life story I began this paper is on yet another. All three of these routes reflect and incorporate Malawi’s history as a predominantly rural but urbanising country, unevenly integrated into a global economy and one that has been at the receiving end of global health interventions. These routes may of course interact and intersect, and all three are influenced by a range of relatively new environmental exposures (including air pollution and pesticide use), the full measure and impact of which we have yet to grasp. I have argued in this paper that a reductive version of ‘metabolic syndrome’ may obscure more than it illuminates in this context, but a more expansive and complex concept of metabolism may still be good to think with. The new science of metabolism, and the recent rapprochement between the social and biological sciences point the way to more nuanced and more accurate understandings of history, biology and epidemiological change (Lock and Nguyen 2010; Meloni et al. 2016).

## Footnotes

1 Original as Bernard’s insights were, they can also be connected with a much longer history of ideas about ‘balance’ within the body, which are a feature of many theories of the body across the world (Nutton 1993).

2 The concept of metabolism also has a lively existence in the environmental movement (Martinez-Aller et al. 2010).

3 Dyslipidemia refers to the elevation of plasma cholesterol, triglycerides or both, or a low high-level density lipoprotein level, thought to contribute to the development of atherosclerosis.

4 For other African examples of this more general phenomenon, see Susan Reynolds Whyte’s account of hypertension and diabetes in Uganda and Julie Livingston’s study of cancer in Botswana. (Whyte 2015; Livingston 2012). On the uses and abuses of ‘metrics’ in global health, see Adams (2016).

5 On the history of the related category of ‘chronic disease’ see Weisz (2014).

6 For an unusually sceptical account and a strongly worded cautionary note on the data, see Roth and Murray (2013).

7 For a recent re-evaluation see the Special Issue of Global Health Action (2014). For an incisive critique of the limitations of the model as applied to Africa see Defo (2014), in the same volume. On the framing of non-communicable diseases in Africa see Whyte (2012).

8 See, for example, the important work on the “nutrition transition” by Barry Popkin and his colleagues: Popkin (2001), Popkin and Gordon-Larsen (2004), Popkin et al. (2012). For South Africa’s food economy and its meanings, see Cousins (2015), Crush et al. (2011), Chopra (2003).

9 On health metrics in relation to maternal mortality in Malawi see the insightful work of medical anthropologist Claire L. Wendland (Wendland 2016).
